# Combining Digital and Molecular Approaches Using Health and Alternate Data Sources in a Next-Generation Surveillance System for Anticipating Outbreaks of Pandemic Potential

**DOI:** 10.2196/47673

**Published:** 2024-01-09

**Authors:** Pablo Ivan P Ramos, Izabel Marcilio, Ana I Bento, Gerson O Penna, Juliane F de Oliveira, Ricardo Khouri, Roberto F S Andrade, Roberto P Carreiro, Vinicius de A Oliveira, Luiz Augusto C Galvão, Luiz Landau, Mauricio L Barreto, Kay van der Horst, Manoel Barral-Netto, ÆSOP Collaborating Teams

**Affiliations:** 1 Center for Data and Knowledge Integration for Health (CIDACS), Instituto Gonçalo Moniz Fundação Oswaldo Cruz (Fiocruz) Salvador Brazil; 2 The Rockefeller Foundation New York, NY United States; 3 Núcleo de Medicina Tropical Universidade de Brasília Brasília Brazil; 4 Escola Fiocruz de Governo Fundação Oswaldo Cruz (Fiocruz) Brasília Brazil; 5 Medicine and Precision Public Health Laboratory (MeSP2) Instituto Gonçalo Moniz Fundação Oswaldo Cruz (Fiocruz) Salvador Brazil; 6 Physics Institute Federal University of Bahia Salvador Brazil; 7 Centro de Relações Internacionais em Saúde (CRIS) Fundação Oswaldo Cruz (Fiocruz) Rio de Janeiro Brazil; 8 Department of Civil Engineering (COPPE) Federal University of Rio de Janeiro Rio de Janeiro Brazil

**Keywords:** data integration, digital public health, infectious disease surveillance, pandemic preparedness, prevention, response

## Abstract

Globally, millions of lives are impacted every year by infectious diseases outbreaks. Comprehensive and innovative surveillance strategies aiming at early alert and timely containment of emerging and reemerging pathogens are a pressing priority. Shortcomings and delays in current pathogen surveillance practices further disturbed informing responses, interventions, and mitigation of recent pandemics, including H1N1 influenza and SARS-CoV-2. We present the design principles of the architecture for an early-alert surveillance system that leverages the vast available data landscape, including syndromic data from primary health care, drug sales, and rumors from the lay media and social media to identify areas with an increased number of cases of respiratory disease. In these potentially affected areas, an intensive and fast sample collection and advanced high-throughput genome sequencing analyses would inform on circulating known or novel pathogens by metagenomics-enabled pathogen characterization. Concurrently, the integration of bioclimatic and socioeconomic data, as well as transportation and mobility network data, into a data analytics platform, coupled with advanced mathematical modeling using artificial intelligence or machine learning, will enable more accurate estimation of outbreak spread risk. Such an approach aims to readily identify and characterize regions in the early stages of an outbreak development, as well as model risk and patterns of spread, informing targeted mitigation and control measures. A fully operational system must integrate diverse and robust data streams to translate data into actionable intelligence and actions, ultimately paving the way toward constructing next-generation surveillance systems.

## Introduction

### Overview

The COVID-19 pandemic has illustrated the limited capabilities of existing surveillance systems worldwide and the need to maximize the use of data to inform public health decisions [1,2]. Among the lessons learned from the pandemic is the critical importance of leveraging early alert warning capabilities to detect and respond to emerging and reemerging pathogens before they cause widespread impact. Unfortunately, existing global surveillance systems are often fragmented, lack coordination and collaboration, and do not prioritize early alerts [3,4]. This has resulted in millions of lives lost due to outbreaks of infectious diseases that usually occur following spillover from contacts at the animal-human interface. Therefore, as governments, civil society, and private entities continue to recover from the losses incurred by the COVID-19 pandemic, it is crucial to sustain the momentum generated by this global crisis and prioritize efforts to elevate health surveillance to the next level.

**Figure 1:**
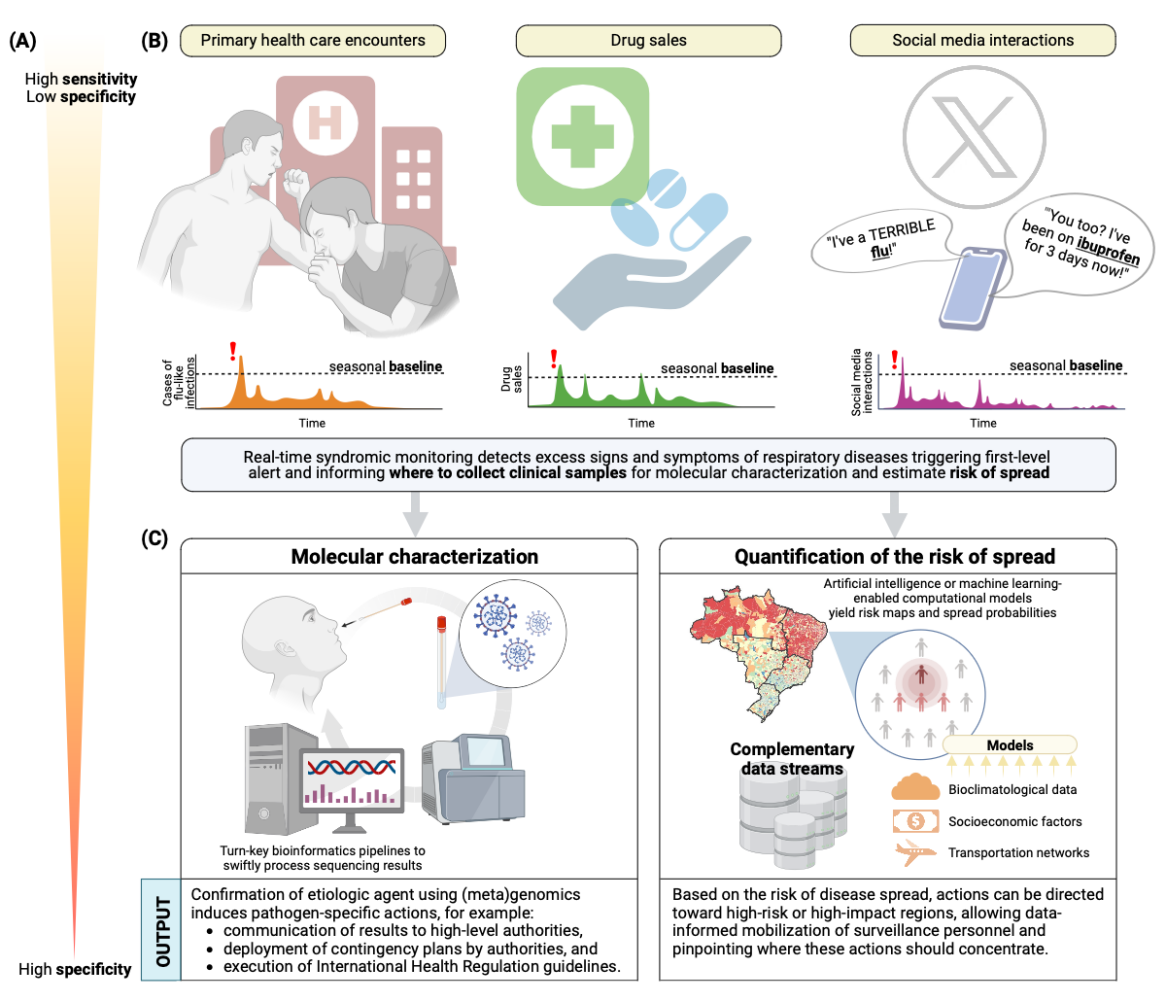
Alert-Early System for Outbreaks with Pandemic Potential (ÆSOP) in overview. (A) The system operates on a continuum that spans from high sensitivity in the initial stages to specific diagnosis, enabled by metagenomics, when required. (B) Continuous syndromic monitoring of health events allows to detect signals in primary health care encounters, over-the-counter sales for selected drug classes, and social media interactions. This monitoring will be performed at the municipal level for Brazil (n=5570 cities). (C) Once a signal is identified, we will use multimodal data integrated into the system, combined with artificial intelligence or machine learning-enabled models, to assess risk and route of outbreak dispersion; in parallel, we will promptly warn health authorities and deploy actions for sample collection and sequencing-based approaches to allow for pathogen characterization.

Developing a system focused on early warning tools is a fundamental requirement to enhance pandemic preparedness and response [2,4]. By investing in disease surveillance systems and building resilient health systems, countries can better prevent, detect, and respond to infectious diseases and other health threats. In this endeavor, we should take advantage of the improvements in several branches of technology and science, in particular digitalization of health routine records, data science, and artificial intelligence modeling, for the rapid gathering and analysis of large amounts of diverse data streams [2,4,5]. Next-generation early-alert warning systems should prioritize sensitivity by integrating various data streams for syndromic surveillance, as opposed to relying on traditional case reports. Additionally, achieving specificity through molecular pathogen characterization is crucial for efficient preemptive actions (Figure 1A). However, the process of collecting and analyzing samples is complex and expensive. To optimize logistics and reduce costs, it is recommended to identify priority areas for sample collection through risk assessment based on syndromic surveillance.

To inform the development of next-generation surveillance systems, here we outline key principles and requirements that should be addressed for creating a comprehensive and effective approach to disease monitoring. We instantiate these principles into the Alert-Early System for Outbreaks with Pandemic Potential (ÆSOP)—a system under development in Brazil that aims to improve the surveillance capacity and pandemic preparedness of this country.

### Preparing for the Next Pandemic by Harnessing the Power of Data

Many countries, Brazil included, have now established national digital systems that capture routine health information, such as clinical consultations at the primary health care (PHC) level, hospital admissions, and drug prescriptions, and the COVID-19 pandemic has also accelerated integration of these digital systems within health systems and networks [6-8]. The next generation of surveillance systems should leverage this existing and emerging rich data landscape and integrate health routine databases with alternate data streams.

In ÆSOP, early warning will be achieved by monitoring of clinical-related data, which enables the mining of potentially anomalous patterns, such as unexpected surges of respiratory diseases outside the expected seasonality or in atypical age groups or locations (Figure 1B). More specifically, PHC weekly visits coded by reason for encounter, as well as drug sales related to acute respiratory syndromes and social media interactions will be monitored. Brazil’s national PHC database, SISAB (Information System for Primary Health Care; in Portuguese, *Sistema de Informação em Saúde para a Atenção Básica*), harbors data on the majority of public-funded PHC encounters in the country [9], coded by either the International Classification of Diseases or the International Classification of Primary Care, with 5535 out of 5570 municipalities reporting to SISAB in 2020, corresponding to 98.5% of the total Brazilian population for that year. Complementary, drug sales data encompasses approximately 95% of all drug sales in Brazil. Continuous monitoring of clinical-related data streams in ÆSOP will enable the system to detect shifts in patterns, which can signal potential outbreaks. By issuing anticipatory warnings to health authorities, the system aims to provide early and timely alerts, as severe cases are not expected to be reported until days or weeks after an increase in mild cases seeking PHC assistance [1,10]. A proof-of-concept evaluating the early-warning capability performance of the system was conducted in Brazil, totaling over 589 million patient visits to PHC facilities between 2017-2020 in 97% of Brazilian municipalities [11]. Of these visits, 4.24% were associated with upper respiratory infections, and results showed that rises in patient consultation in PHC encounters for respiratory disease were able to anticipate increases of severe acute respiratory syndromes, with geographical variabilities [11]. In a retrospective analysis conducted with data from Bahia, a state with 14.8 million inhabitants in the Northeast region, PHC syndromic monitoring was able to detect COVID-19 entry in 20 out of 21 regions of the state at least 1 week before the rise in COVID-19 cases, showing feasibility for use of the system [12]. The system can also be adapted to the monitoring of other syndromes, such as dengue-like illnesses, by reparametrizing the codes for reason for encounter and drugs used to treat these diseases. This flexibility by design is a key feature that enables the system to be customized to the specific needs of different regions and populations.

Different modeling frameworks were developed and applied to health data aiming at the early detection of outbreaks [13-17], including statistical, mathematical, and computational methods. Although some of them are in use in the routine of health surveillance agencies [15,16], to the best of our knowledge none has been adopted as a gold standard of undoubtedly effectiveness. However, all methods have limitations, and further developments are still needed in this field [2-4]. The proposal described herein aims to combine several approaches in a systemic logical approach combining their strengths. Table 1 presents general features of traditional health surveillance in comparison to what we envision would make a next-generation surveillance system (exemplified by ÆSOP) that overcomes identified deficiencies which hamper pandemic preparedness and response.

**Table 1:** Comparison of key features in traditional disease surveillance systems with next-generation approaches.

Characteristic	Traditional surveillance	Next-generation surveillance
Data sources	Largely focused on notifiable diseases, clinical and laboratory reports, but can also include sentinel surveillance, registries, surveys, and administrative data systems	Expands these streams to include syndromic data, drug sales, social media, rumors on news outlets and in the digital world, and can be fit to distinct syndromes and to accommodate country-specific data assets
Data integration	Limited integration of different data streams	Integrates diverse data streams, enabling a comprehensive view
Data sharing	Limited sharing of data between countries and agencies	Encourages data sharing within a federated approach, allowing for global data collaboration without compromising sovereignty
Interoperability	Limited interoperability between different health agencies and stakeholders due to lack of standards	Promotes collaboration among various entities by leveraging standardized vocabularies or ontologies
Early alerting	Typically relies on case reports and may lead to delayed detection	Uses syndromic data for early alerting, enhancing timely outbreak detection
Flexibility	Often lacks adaptability for different syndromes or regions	Designed with flexibility to target various syndromes
Molecular analysis (pathogen specificity)	Focuses on traditional laboratory-based pathogen analysis	High-throughput metagenomics for rapid pathogen characterization at specific regions informed by syndromic monitoring

This approach should overcome current public health response limitations, as digital health has been recognized as a critical element [18] and an emerging discipline to prepare for, prevent, and respond to pandemics [1,10], while the syndromic surveillance approach will allow for greater sensitivity [19]. It is also of great importance that this monitoring tool is built over existing systems, as we aim for cost-effectiveness, and no additional burden should be added to already overwrought health systems. The system aims to complement traditional surveillance methods, which in Brazil rely mainly on the mandatory notification of suspected cases of priority diseases listed by the Ministry of Health, a usually paper-based process that often incurs in notification delays.

### Combining Digital and Molecular Approaches for Achieving Both Sensitivity and Specificity

Genomics-enabled pathogen discovery has been proposed as a game changer for infectious disease surveillance, which some—optimistically—refer to as “not yet a panacea” [20]. The genomics component of ÆSOP, however, leverages sequencing power to provide pathogen specificity for the system at a later stage, rather than initially. By building upon the existing digitally informed streams of syndromic surveillance, which allows for “broad, but swift” monitoring, and using its output to pinpoint areas for the pathogen identification strategy continuously, ÆSOP aims to equate the costs associated with real-time event monitoring paralleled with targeted genomics. With further reduction of DNA sequencing costs in developing countries, metagenomics may become a key epidemiological tool for rapid pathogen identification.

Alerts generated by clinical-related data will be used internally to activate site-directed genomic analysis for pathogen identification. This will allow for optimizing the logistic strategy for obtaining and analyzing samples. For instance, once a signal of a potential outbreak is identified, field teams will collect clinical samples that undergo molecular characterization for pathogen identification over a small area (Figure 1C). A major challenge in the system is the timely transportation of samples to the processing sites. In large countries with logistic hindrances, it is important to have decentralized sequencing sites for shortening transportation delays. ÆSOP will leverage and use the capillarity of the existing Brazilian Genomic Network facilities, such as that induced by Fiocruz during the COVID-19 pandemic [21], the National Network of Health Surveillance Laboratories, and others to speed-up the identification of circulating pathogens. Similar networks have been established in other countries as well, including the COVID-19 Genomics UK Consortium [22], the Indian SARS-CoV-2 Genomic Consortium [23], and the Africa Pathogen Genomics Initiative [24], among others. These initiatives have proven to be valuable in the response against SARS-CoV-2, providing critical insights into the spread and evolution of the virus. As the number of COVID-19 cases continues to decrease in many countries, it is vital that these networks are strengthened and expanded to ensure that they can be used as an essential component of next-generation surveillance systems, allowing for identification of pathogens behind potential outbreaks.

This integrated approach combines both data- and hypothesis-driven disease surveillance approaches, enabling quick identification of anomalies through syndromic surveillance using a combination of bona fide health data and alternative data streams (data-driven approach); once anomalies are identified, targeted sample collection and modeling (hypothesis-driven approach) can be used to confirm the outbreak and determine appropriate actions. To generate risk maps, computational models will integrate socioeconomic, environmental, and mobility and transportation data. Additionally, mathematical models coupled with artificial intelligence and machine learning methods will enable the assessment of risk and routes of outbreak spread (Figure 1B and 1C).

### Data Security, Privacy, and Trust

Another unique feature of ÆSOP is the use of a defined methodology for the collection, linkage, and use of relevant and appropriate data. We will use, extract, transform, and load protocols [25] for capturing and standardizing data from different sources, and machine learning methods for feature engineering and longitudinal multimodal data integration. To ensure the security and privacy of data, ÆSOP will follow strict legal and ethical guidelines established by the Center of Data and Knowledge Integration for Health, which has already implemented robust rules and regulations for working with health data, based on global best practices [26]. This approach will ensure that ÆSOP not only provides early warning surveillance for outbreaks of epidemic or pandemic potential but also protects the confidentiality of individuals’ health information.

To ensure the responsible use of data, all information collected will be subject to rigorous digital curation procedures and preserved according to established standards [26]. The resulting deidentified and aggregated data will be made available to health authorities and the scientific community through an application programming interface, allowing for integration with other relevant data sources. This comprehensive set of spatially integrated health, environmental, social, and contextual data has the potential to be a valuable resource for research and other purposes beyond outbreak surveillance.

### Fit for Purpose

An essential aspect of ÆSOP is its development in close collaboration with stakeholders and end users, ensuring its adoption by health authorities and successful implementation in mitigating and preventing outbreaks. We have collected feedback and identified key requirements from end users to inform rapid response, by convening workshops and focused meetings. Ultimately, these will lead to creating and fostering local/regional communities of practice for surveillance that will involve end users and other stakeholders in the development and implementation of surveillance systems, inspired by previous experiences such as the US Centers for Disease Control and Prevention’s National Syndromic Surveillance Program [27]. These communities will provide a platform for sharing experiences, challenges, and best practices in surveillance, and will serve as a valuable source of feedback for improving the system. By engaging with end users in this way, we aim to create a sense of ownership and investment in the system, which is crucial for ensuring its long-term success and sustainability through adoption.

### Balancing Surveillance Data Sharing Needs With Data Sovereignty Concerns Using a Federated Approach

**Figure 2:**
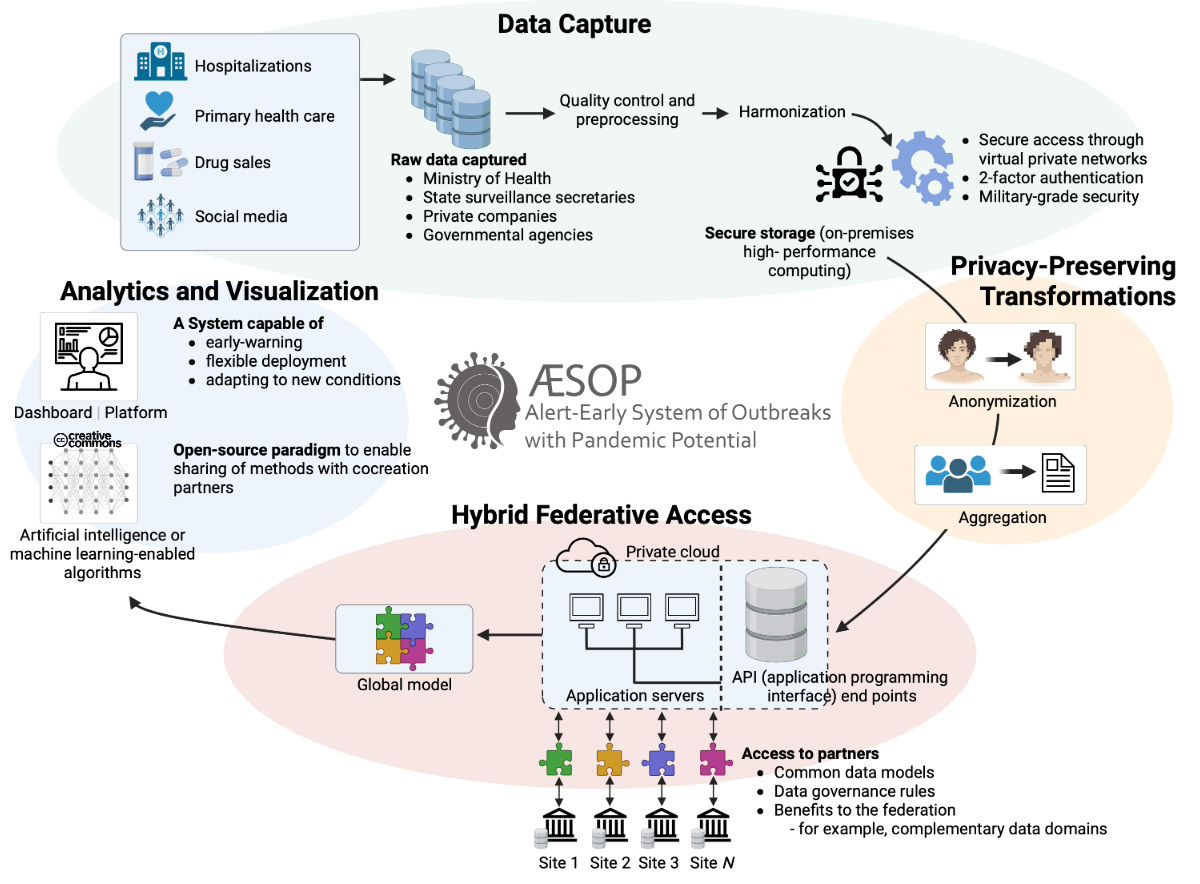
A federated approach to surveillance. After capture and integration of health data, transformations aimed at removing personally identifiable information and aggregation are performed. This data can be transferred to secure environments to allow for the training of models and analytics, and more refined, global models can be attained with federated learning approaches that parametrize the global model based on feedback from models trained at other sites without transfer of raw data among sites. The monitoring of clinical-related data and modeling outputs will be made available as a platform to stakeholders including health surveillance authorities.

A critical lesson learned from responding to the COVID-19 pandemic is the need to build trust between governments, organizations, and societies to facilitate the early sharing of accurate information for responsive global health surveillance [2,4]. However, individuals’ privacy and data sovereignty concerns cannot be ignored. While balancing these necessities is not trivial, we understand that finding a solution to safely share information to guide decisions and provide global health security is crucial. In this sense, federated approaches represent a feasible solution that balances the need for data sharing with the need to protect data privacy and sovereignty (Figure 2). By allowing data owners to maintain control over their data, a federated system can help encourage collaboration between organizations, with sharing occurring only on a need-to-know basis. This is particularly relevant in health surveillance, where potentially sensitive data are involved.

A subset of the federated approach, federated learning enables independently built models to be trained on distributed data sets without sharing of raw data; then, an updated model is sent back to a central server to be aggregated with other models, and by iteratively exchanging parameters a globally refined model emerges [28]. This allows for the development of a more robust and accurate model while preserving data privacy. Combined, federated approaches provide a powerful solution for meeting the swift exchange of information under the International Health Regulations while also ensuring compliance with local legislation on individual privacy. Common data models and standards are necessary to ensure that data can be exchanged, integrated, and analyzed effectively across different sites [29].

### Conclusions

The COVID-19 pandemic, and those before it, evidenced the need for substantial improvements in pandemic preparedness and response, globally. We presented key features and essential building blocks of the ÆSOP system—a system being developed with a combination of data-driven and hypothesis-driven infectious disease surveillance approach for early-alert identification of areas with significant risk of reemergence of infectious disease outbreaks. ÆSOP aims to accurately anticipate an outbreak when compared to currently in-place surveillance systems. Moreover, ÆSOP’s reports should enable precision public health strategies, such as targeted early outbreak mitigation and response strategies, as well as site-directed sample collection for pathogen molecular characterization, thus improving logistics and enhancing responses. To ensure that next-generation surveillance systems will properly meet public health needs, and to obtain a broader perspective of possibilities and bottlenecks, active engagement and cocreation processes with stakeholders, end users, and multidisciplinary experts will be essential and key to building and refining enhanced surveillance systems. Final, we are aware of the revision of the International Health Regulations and the preparation of the Pandemic Treaty, for eventual adaptation of ÆSOP to these rules.

## Appendix

Multimedia Appendix 1 List of members of the ÆSOP Collaborating Teams.

## Abbreviations

ÆSOP: Alert-Early System for Outbreaks with Pandemic Potential
PHC: primary health care
SISAB: Sistema de Informação em Saúde para a Atenção Básica; in English, Information System for Primary Health Care

## Data Availability

Data sharing is not applicable to this article as no data sets were generated or analyzed during this study.
