# Supplementary material for: Combining Digital and Molecular Approaches Using Health and Alternate Data Sources in a Next-Generation Surveillance System for Anticipating Outbreaks of Pandemic Potential
Source: JMIR Public Health Surveill. 2024 Jan 9;10:e47673. doi: 10.2196/47673 (PMC10806444; doi:10.2196/47673)
Supplement: Multimedia Appendix 1 [file publichealth_v10i1e47673_app1.docx]

# Multimedia Appendix 1 - List of members of the ÆSOP Collaborating Teams

| **Member** | **Affiliation** |
| --- | --- |
| Adriano de Oliveira Vasconcelos | Civil Engineering (COPPE), Federal University of Rio de Janeiro, Rio de Janeiro, Brazil |
| Alexandre Gonçalves Evsukoff | Civil Engineering (COPPE), Federal University of Rio de Janeiro, Rio de Janeiro, Brazil |
| Alvaro Luiz Gayoso de Azeredo Coutinho | Civil Engineering (COPPE), Federal University of Rio de Janeiro, Rio de Janeiro, Brazil |
| Maria Célia S Lopes Cunha | Civil Engineering (COPPE), Federal University of Rio de Janeiro, Rio de Janeiro, Brazil |
| Diogo Antonio Tschoeke | Biomedical Engineering Program - COPPE, Federal University of Rio de Janeiro (UFRJ), Rio de Janeiro, Brazil |
| Fabiano L Thompson | Laboratory of Microbiology, Biology Institute, Federal University of Rio de Janeiro (UFRJ), Rio de Janeiro, Brazil and Center of Technology - CT2, SAGE-COPPE, Federal University of Rio de Janeiro (UFRJ), Rio de Janeiro, Brazil |
| Fabio Hochleitner | Civil Engineering (COPPE), Federal University of Rio de Janeiro, Rio de Janeiro, Brazil |
| Felipe Gomes Naveca | Leonidas and Maria Deane Institute, Oswaldo Cruz Foundation, Terezina Street, Manaus, Amazonas, Brazil |
| Gerson Gomes Cunha | Civil Engineering (COPPE), Federal University of Rio de Janeiro, Rio de Janeiro, Brazil |
| Malu Grave | Civil Engineering (COPPE), Federal University of Rio de Janeiro, Rio de Janeiro, Brazil  Center for Data and Knowledge Integration for Health (CIDACS), Instituto Gonçalo Moniz, Fundação Oswaldo Cruz, Salvador, Brazil |
| Marcelo Ferreira da Costa Gomes | Programa de Computação Científica, Fundação Oswaldo Cruz, Rio de Janeiro, Brazil |
| Marcos Ennes Barreto | Department of Statistics, London School of Economics and Political Science, London, UK and Center for Data and Knowledge Integration for Health (CIDACS), Instituto Gonçalo Moniz, Fundação Oswaldo Cruz, Salvador, Brazil |
| Pedro Milet Meirelles | Institute of Biology, Federal University of Bahia, Salvador, Brazil and National Institute for Interdisciplinary and Transdisciplinary Studies in Ecology and Evolution (IN-TREE), Salvador, Brazil |
| Pilar Veras Fiorentino | Center for Data and Knowledge Integration for Health (CIDACS), Instituto Gonçalo Moniz, Fundação Oswaldo Cruz, Salvador, Brazil  Institute of Biomedical Science, University of São Paulo, São Paulo, Brazil |
| Priscilla Normando | Center for Data and Knowledge Integration for Health (CIDACS), Instituto Gonçalo Moniz, Fundação Oswaldo Cruz, Salvador, Brazil |
| Thiago Cerqueira Silva | Faculdade de Medicina, Universidade Federal da Bahia (UFBA)  Center for Data and Knowledge Integration for Health (CIDACS), Instituto Gonçalo Moniz, Fundação Oswaldo Cruz, Salvador, Brazil |
| Viviane S Boaventura | Instituto Gonçalo Moniz, Fundação Oswaldo Cruz, Salvador, Brazil  Universidade Federal da Bahia, Salvador, Brazil |
